# An Atypical Cutaneous Metastasis in a Case of Clear Cell Renal Carcinoma

**DOI:** 10.7759/cureus.30722

**Published:** 2022-10-26

**Authors:** Tomas Escobar Gil, Sara Saldarriaga Santamaría, Juan J Del Valle Saavedra, Ana M Mejía Giraldo, Elsa B Peña Zúñiga

**Affiliations:** 1 Medicine, Universidad CES, Medellín, COL; 2 Dermatology, Universidad CES, Medellín, COL; 3 Dermatology, Hospital General de Medellín Luz Castro de Gutiérrez, Medellín, COL

**Keywords:** kidney biopsy, clear renal cell carcinoma, treatment, metastasis, cutaneous

## Abstract

Clear cell renal cell carcinoma (ccRCC) comprises most renal cell carcinoma (RCC) cases, with its incidence increasing in recent years. Metastases are most commonly found in the lungs, bones, liver, and brain. However, few patients present with cutaneous metastases, which are usually associated with poor outcomes. We present the case of a 52-year-old man with ccRCC and skin metastasis. Our aim was to highlight the variability in the presentation of cutaneous metastasis of ccRCC. Clinicians should be aware of the various manifestations and possible locations of RCC skin metastases to better identify these lesions and further guide treatment.

## Introduction

Kidney cancer is the ninth most common malignancy in the United States [1]. Clear cell renal cell carcinoma (ccRCC) comprises 75% of these cases, with incidence increasing in recent years. It accounts for approximately 2% of cancer diagnoses and deaths worldwide. Developing countries, like Colombia, have fewer incidence rates, with less information on poor-income areas [2].

Metastases are most frequently seen in the lungs, bones, liver, and brain [1]. Hematogenous spread may result in an unusual metastatic pattern such as muscular or cutaneous disease [1]. As many as 3% of patients present with cutaneous metastases, which usually are associated with a poor outcome [3,4]. Skin metastases of internal tumors, including kidney cancer, have also increased in the last decade [4].

Frequent clinical manifestations of cutaneous metastasis include painless red nodules and plaques [4]. Ulcers, and pink papules, among several other presentations, including inflammatory, cicatricial, and bullous lesions, have been reported but are not as frequent [4,5]. Blue-colored presenting lesions have been described as a unique presentation of renal cancer, liver cancer, and neuroblastomas [4].

When a metastatic skin lesion is suspected, a thorough work-up including a skin biopsy with appropriate histologic stains should be performed [4]. A biopsy can also be used to establish the primary malignancy if unknown, as the histopathologic appearance of the metastatic tissue may mimic the primary tumor [5].

We found other case reports of cutaneous involvement of renal cell carcinoma (RCC). In most of these reports, the presenting lesion consisted of a nodule or a pedunculated lesion, which is the classical presentation of skin metastases of RCC [1,3,6-10].

We present the case of a 52-year-old man with an atypical presentation of cutaneous metastasis of ccRCC and highlight the importance of considering the variability of clinical manifestations and locations of ccRCC cutaneous metastases.

## Case presentation

**Figure 1:**
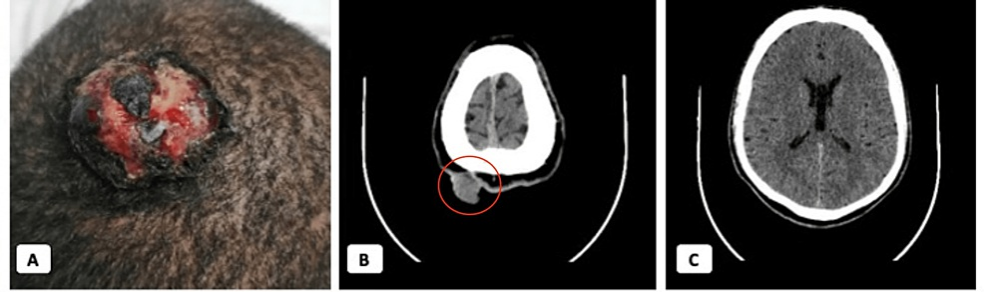
Clinical appearance of the lesion and imaging. (A) An exophytic mass located in the coronal region of the scalp, with bleeding stigmata. (B) Contrast tomography (CT) of the skull showing an exophytic mass on the scalp of the right parietal region with deep subgaleal involvement. The involved area is marked with a red circle. (C) No periosteal infiltration or intracranial involvement was observed.

We present the case of a 52-year-old African American male with a past medical history of ccRCC of the right kidney, ISUP grade 4, TNM stage IV: T2N0M1, with metastases to lung and bone, who had undergone radical nephrectomy of the right kidney and five cycles of treatment with pembrolizumab and axitinib. He presented to the dermatology service with a four-month-old lesion on the scalp associated with bleeding and headache. On physical examination, a 3-cm pedunculated, ulcerated mass with bleeding stigmata was noted in the coronal region of the scalp (Figure 1A). Computed tomography (CT) of the skull was ordered (Figures 1B, 1C), which revealed an exophytic mass on the scalp of the right parietal region, highly vascularized, measuring 20 x 30 x 30 mm, with deep subgaleal involvement, without periosteal infiltration or intracranial involvement. With these results, resection of the scalp tumor with coverage using a diadem flap was planned and performed by plastic surgery.

**Figure 2:**
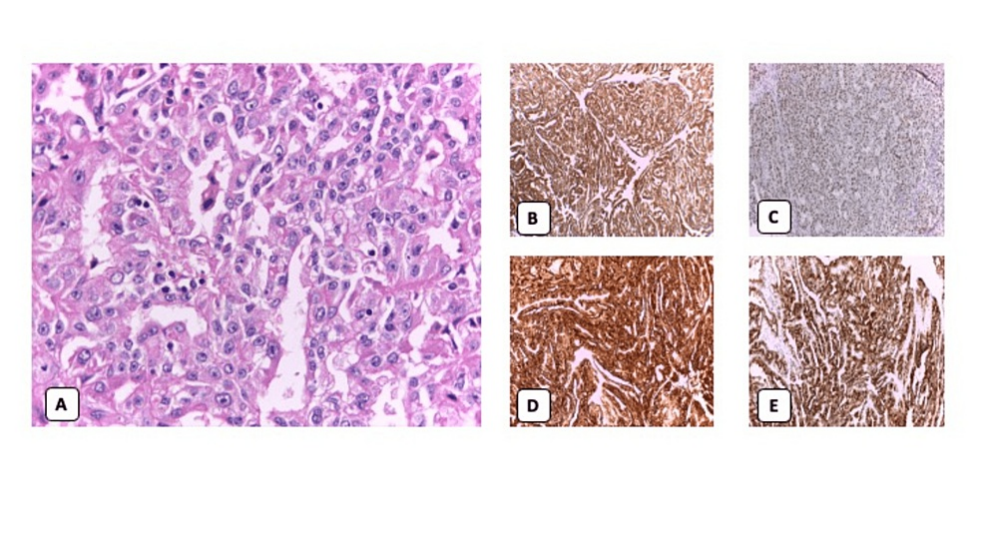
Histopathological findings and immunohistochemistry. (A) Hematoxylin and eosin stain of the metastatic cutaneous lesion with 40X magnification: tumorous lesion in the dermis and subcutaneous cellular tissue, with extensive ulcerated areas, foci of lympho-histio-plasmacytic infiltrate, necrosis, and hemorrhage. Rows, sheets, and tubular structures containing cells with abundant clear and eosinophilic cytoplasm, vesicular chromatin nuclei with prominent nucleolus, and frequent mitotic figures were observed. Findings are marked with a red circle for identification. Immunohistochemical study: (B) cocktail of cytokeratin+, (C) CD10+, (D) PAX 8+, (E) renal cell carcinoma marker+.

The histopathological study revealed a tumorous lesion that occupied the dermis and subcutaneous cellular tissue, with extensive ulcerated areas, foci of necrosis, and hemorrhage (Figure 2A). The specimen was composed of rows, sheets, and tubular structures containing cells with abundant clear and eosinophilic cytoplasm, vesicular chromatin nuclei, prominent nucleolus, and frequent mitotic figures (up to 4 per high-power field). There were foci of lympho-histio-plasmacytic infiltrate. The resection margins were free of tumor involvement.

Regarding the immunohistochemical study, a cocktail of CK+, CD10+, PAX 8+, RCC marker+, CAIX-, CD34-, CK7-, and CK20- was obtained (Figures 2B-2E). With these findings, the diagnosis of skin involvement of ccRCC was made.

Six months later, during follow-up, a new CT showed no new metastases and the patient continued outpatient oncology care.

## Discussion

Cutaneous metastases are not a common finding in the setting of ccRCC [1-7]. They are usually seen in the final stages of the disease but can be found at any stage and at any time [7]. Our patient’s time of presentation is consistent with the timing described in the literature, as he had a stage IV ccRCC.

Some typical sites of cutaneous metastases are the chest and the abdomen due to anatomic proximity to the kidneys, and the scalp due to lymphohematogenous dissemination, as noted above [7]. Our case shows these features; however, its presentation is unique in the sense that the characteristics of the lesion are atypical. The painful, ulcerated nature of the lesion and the hemorrhage are not consistent with the most common descriptions of metastatic ccRCC skin lesions in other reports we found [1,3,6-20]. The findings of such reports are comprised in Table 1.

**Table 1:** Literature reports about skin metastasis of clear cell renal carcinomas

Article name	Author	Anatomic location of cutaneous metastasis	Lesion description	Age	Gender
Metastatic Clear Cell Renal Cell Carcinoma to the Forearm Without Identifiable Primary Renal Mass [1]	Walton et al.	Right forearm	Painless enlarging mass	52	Male
Cutaneous Metastases in Renal Cell Carcinoma: a Systematic Review and a Case Report [3]	Ohlsson et al.	Left flank	Painless cutaneous lesion	76	Female
Cutaneous Metastases and Long-Term Survival of a Patient With Clear Cell Renal Carcinoma [6]	Vilaca et al.	Right flank	Painful violaceous skin lesions	59	Male
A Pedunculated Skin Lesion in a Case of Clear Cell Renal Carcinoma [7].	Kesireddy et al.	Submandibular region	Isolated pedunculated nodule	39	Male
Cutaneous Metastasis of Renal Cell Carcinoma to the Cheek: A Case Report and Literature Review [8]	Silver et al.	Left cheek	Rapidly growing mass, nonfriable nodules with no overlying skin changes	72	Male
Rare Abdominal Cutaneous Presentation of Clear Cell Renal Cell Carcinoma: A Case Report [9]	Osen and Warren	Left iliac fossa	Friable, well-demarcated, red-purple lesion with minimal surrounding erythema	69	Male
Clear Cell Kidney Carcinoma Revealed by Cutaneous and Sinus Metastases: About a Case and Literature Review [10]	Ndounga et al.	Right scalp	Painless enlarging mass	70	Male
Skin Metastasis of Renal Cell Carcinoma [11]	Mitomi et al.	Right lower abdomen	Cutaneous nodule	68	Female
Cutaneous Metastasis of Renal Cell Carcinoma: A Report of Two Cases [12]	Tadashi et al.	Neck	Hemangioma	84	Male
Cutaneous Metastasis of Renal Cell Carcinoma: a Report of Two Cases [12]	Tadashi et al.	Thigh	N/A	66	Male
Cutaneous Metastasis of Renal Cell Carcinoma: Case Report and Review of the Literature [13]	Fernández-Rueda et al.	Anterior neck	Subcutaneous neoformation of a hard, mobile consistency, not adhered to deep planes, with well-defined borders, red-violet coloration, and telangiectasias	66	Male
Cutaneous Metastases of Renal Carcinoma in the Toes [14]	Elfatoiki et al.	First and fourth toes of the left foot	Angiomatous subcutaneous nodule on the first toe, well limited, and on the fourth toe angiomatous-like nodule with a budding surface that bled on contact	64	Female
Three Synchronous Atypical Metastases of Clear Cell Renal Carcinoma to the Maxillary Gingiva, Scalp and the Distal Phalanx of the Fifth Digit: A Case Report [15]	Selvi et al.	Parietal area of the scalp and distal phalanx of the fifth digit of the left hand	N/A	51	Male
Pink Nodule of the Chin: an Unusual Presentation of Metastatic Carcinoma [16].	Chelliah et al.	Right chin	Pink exophytic somewhat friable pedunculated nodule overlying a fluctuant but firm subcutaneous nodule on the right chin	45	Female
Calvarial and Cutaneous Metastasis as the Primary Presentation of a Renal Cell Carcinoma [17]	Jindal et al.	Infraumbilical area	Cutaneous nodule	35	Female
Eyelid Metastasis as the Initial Presentation of a Renal Cell Carcinoma [18]	González et al.	Left inferior eyelid	Solitary ulcerated nodular tumor	77	Male
Cutaneous Metastasis of Genitourinary Origin [19].	Ruiz-Oslé et al.	Left mandibular area	N/A	52	Female
Renal Cell Carcinoma Presenting as a Tumor on the Scalp: A Case Report [20]	Krogerus et al.	Right occipital region	Pulsating, highly vascularized tumor	65	Male

It is clear that the lesion in our case was easily identified because of the pain, size, and location. However, the typical lesion is painless and small, and does not bleed, which makes it easily overlooked by patients and healthcare providers. This raises concern because the asymptomatic nature of presenting lesions may delay identification or treatment of the condition.

Another characteristic that stands out in our case report is the high vascularity found in the CT scan and the bleeding stigmata present during the physical examination, which is paradoxical considering that the patient had undergone five cycles of antiangiogenic therapy with axitinib at the time of presentation. This calls into question the efficacy of the said treatment for cutaneous metastasis of ccRCC given that the patient’s internal metastasis did respond to adjuvant treatment.

Our patient was a surgical candidate and underwent resection of the mass with success, exhibiting no recurrence at six months. This finding reinforces the role of surgical treatment as the gold standard for these lesions. Dismissal of the initial lesion for its unusual characteristics would have been unfortunate considering its complete resolution with treatment. Clinicians should be encouraged to have a low threshold of suspicion when it comes to the inspection of the skin of patients with ccRCC, as cutaneous metastases are heterogeneous in nature and can be easily mistaken for benign conditions [4].

## Conclusions

Cutaneous metastases of ccRCC are infrequent manifestations of the primary tumor. Patients can present with a variety of skin lesions ranging from more commonly encountered ones, such as nodules and plaques, to rare ones, such as ulcerated exophytic lesions. It is crucial that general practitioners, oncologists, and dermatologists perform detailed skin examinations in patients with internal tumors, especially ccRCC. This ensures that possible skin metastases are identified timely and get proper treatment. The metastatic lesion may even be the initial presentation of the internal tumor. Failure to identify these lesions can result in irresectable masses that further increase morbidity and mortality.

## References

[1] Walton J, Li J, Clifton MM, Mori RL, Park AM, Sumfest JM (2019). Metastatic clear cell renal cell carcinoma to the forearm without identifiable primary renal mass. Urol Case Rep.

[2] Sung H, Ferlay J, Siegel RL, Laversanne M, Soerjomataram I, Jemal A, Bray F (2021). Global Cancer Statistics 2020: GLOBOCAN estimates of incidence and mortality worldwide for 36 cancers in 185 countries. CA Cancer J Clin.

[3] Ohlsson R, Geertsen L, Berge Stuveseth S, Lund L (2019). Cutaneous metastases in renal cell carcinoma: a systematic review and a case report. Scand J Urol.

[4] Choate EA, Nobori A, Worswick S (2019). Cutaneous metastasis of internal tumors. Dermatol Clin.

[5] Jaros J, Hunt S, Mose E, Lai O, Tsoukas M (2020). Cutaneous metastases: a great imitator. Clin Dermatol.

[6] Vilaça M, Braga F, Mesquita A (2022). Cutaneous metastases and long-term survival of a patient with clear cell renal carcinoma. Cureus.

[7] Kesireddy M, Correa A, Correa R, Venkatesan R (2019). A pedunculated skin lesion in a case of clear cell renal carcinoma. Cureus.

[8] Silver E, Roudakova K, Bial N, Daniel D (2021). Cutaneous metastasis of renal cell carcinoma to the cheek: a case report and literature review. Am J Case Rep.

[9] Osen ER, Warren AY (2019). Rare abdominal cutaneous presentation of clear cell renal cell carcinoma: a case report. Urology.

[10] Ndounga E, Peko JF, Mbon JB (2018). [Clear cell kidney carcinoma revealed by cutaneous and sinus metastases: about a case and literature review] [Article in French]. Pan Afr Med J.

[11] Mitomi T, Kawahara T, Nomura S (2020). Skin metastasis of renal cell carcinoma. Case Rep Oncol.

[12] Tadashi T (2012). Cutaneous metastasis of renal cell carcinoma: a report of two cases. Int J Clin Exp Pathol.

[13] Fernández-Rueda P, Ruiz-López P, Ramírez-Negrín MA, Fuentes-Suárez A, Toussaint-Caire S, Vega-Memije ME (2015). Metástasis cutánea de carcinoma de células renales (MCCCR): reporte de caso y revisión de la literatura. Gac Med Mex.

[14] Elfatoiki FZ, Chiheb S, Moukhlissi M, Marnissi F, Benchikhi H (2013). [Cutaneous metastases of renal carcinoma in the toes]. Ann Dermatol Venereol.

[15] Selvi F, Faquin WC, Michaelson MD, August M (2016). Three synchronous atypical metastases of clear cell renal carcinoma to the maxillary gingiva, scalp and the distal phalanx of the fifth digit: a case report. J Oral Maxillofac Surg.

[16] Chelliah P, Shah KM, Vandergriff T, Nijhawan RI (2021). Pink nodule of the chin: an unusual presentation of metastatic carcinoma. Dermatol Online J.

[17] Jindal T, Sinha RK, Mukherjee S, Karmakar D (2014). Calvarial and cutaneous metastasis as the primary presentation of a renal cell carcinoma. BMJ Case Rep.

[18] Gonzalez F, Abalo-Lojo JM, Suarez-Peñaranda JM, Caneiro-Gómez J (2015). Eyelid metastasis as the initial presentation of a renal cell carcinoma. Urology.

[19] Ruiz-Oslé V, Crespo-Atín S, Ruiz-Oslé S (2019). Metástasis cutánea de origen genitourinario. Arch Esp Uro.

[20] Krogerus C, Svenning M, Pilt AP, Trøstrup H (2020). Renal cell carcinoma presenting as a tumor on the scalp: A case report. Int J Surg Case Rep.

